# Measurement Properties of the Arabic Pain Self-Efficacy Questionnaire in Individuals with Neck Pain

**DOI:** 10.3390/healthcare14183099

**Published:** 2026-09-20

**Authors:** Abdulrahman M. Alsubiheen, Mishal M. Aldaihan, Ali H. Alnahdi

**Affiliations:** Department of Rehabilitation Sciences, College of Applied Medical Sciences, King Saud University, P.O. Box 10219, Riyadh 11433, Saudi Arabia

**Keywords:** pain self-efficacy, neck pain, patient-reported outcome measure, psychometrics, reliability, validity, cognitive factor

## Abstract

**Background/Objective:** Pain self-efficacy is an important determinant of pain-related disability and recovery in individuals with musculoskeletal disorders. Although the Arabic Pain Self-Efficacy Questionnaire (PSEQ) has demonstrated satisfactory psychometric properties in individuals with chronic low back pain, its measurement properties have not been established in individuals with neck pain. This study evaluated the measurement properties of the Arabic PSEQ in individuals with neck pain. **Methods:** A prospective cohort study was conducted in 118 Arabic-speaking individuals with neck pain recruited from multiple clinics. The Arabic PSEQ, Neck Disability Index (NDI), RAND-36 Health Survey, and Numeric Pain Rating Scale (NPRS) were completed by the participants. Structural validity was examined using exploratory factor analysis. Internal consistency was assessed using Cronbach’s alpha. Test–retest reliability (intraclass correlation coefficient (ICC_2,1_)), measurement error (standard error of measurement (SEM), minimal detectable change (MDC_95_)), and agreement (Bland–Altman analysis) were also examined. Construct validity was assessed by testing predefined hypotheses regarding correlations with comparator measures. **Results:** The analysis revealed a dominant one-factor structure explaining 66.4% of the variance and item loadings ranging from 0.72 to 0.89. Cronbach’s alpha of 0.94 suggested excellent internal consistency. Test–retest reliability was good (ICC_2,1_ = 0.85), with an SEM of 4.38 and MDC_95_ of 12.14. Four of five (80%) predefined construct validity hypotheses were confirmed, demonstrating expected correlations with disability, physical functioning, emotional well-being, and pain intensity. **Conclusions:** The Arabic PSEQ demonstrated a one-factor structure, good test–retest reliability, acceptable measurement error, excellent internal consistency and sufficient construct validity in individuals with neck pain. These findings support its use as a patient-reported outcome measure with good measurement properties for assessing pain self-efficacy in Arabic-speaking individuals with neck pain.

## 1. Introduction

Neck pain is a common musculoskeletal disorder and a leading cause of disability globally. Findings from global studies indicate that greater than 200 million people are affected by neck pain, making it one of the leading contributors to disability worldwide [1,2]. The burden of neck pain extends beyond physical symptoms and includes reduced quality of life, work absenteeism, decreased productivity, and substantial healthcare utilization [3,4,5]. In the Middle East, neck pain represents a significant public health concern, with consistently high prevalence rates and disability burden reported across countries [6]. Given the chronic and recurrent nature of neck pain, identifying factors that influence recovery and long-term outcomes has become an important focus of rehabilitation research.

Among the biopsychosocial factors influencing the course of neck pain, pain self-efficacy has emerged as a key determinant of pain-related outcomes. Pain self-efficacy reflects the confidence in performing daily activities and achieving desired goals despite the presence of pain [7]. Rather than reflecting pain intensity, pain self-efficacy represents the perceived ability to function while experiencing pain. Individuals with high self-efficacy tend to have better physical functioning, lower disability, reduced fear-avoidance behaviors, and better health-related quality of life, whereas lower self-efficacy has consistently been linked to persistent pain, greater disability, psychological distress, and work disability in individuals with musculoskeletal disorders [8,9]. Importantly, prospective studies have identified pain self-efficacy as an independent prognostic factor for recovery, where greater improvements in pain and functional outcomes following rehabilitation are demonstrated by individuals with higher levels of self-efficacy at baseline [10,11]. Consequently, assessment of pain self-efficacy has become increasingly important for identifying patients at risk of poor outcomes and informing individualized treatment.

The Pain Self-Efficacy Questionnaire (PSEQ) is a commonly used pain self-efficacy measure in individuals with persistent pain [7,12]. The PSEQ consists of 10 items that evaluate the confidence in performing a range of activities in spite of pain. PSEQ has been translated into numerous languages and evaluated across a variety of musculoskeletal pain conditions [12]. Previous studies have consistently supported its unidimensional structure, test–retest reliability, internal consistency, and construct validity, supporting its use in both clinical practice and research settings [12]. An Arabic PSEQ has been adapted for Arabic-speaking populations. The first Arabic validation study was conducted in individuals with chronic low back pain and demonstrated sufficient test–retest reliability, internal consistency, and sufficient construct validity [13]. This study also supported the unidimensional structure of the instrument, consistent with the original English version [7]. More recently, an independent Arabic translation and validation study in individuals with chronic low back pain further supported the validity and reliability of the Arabic PSEQ [13]. Collectively, these studies suggest that the Arabic PSEQ is a promising measure for assessing pain self-efficacy among Arabic-speaking individuals with musculoskeletal pain.

However, the available evidence regarding the Arabic PSEQ has been generated exclusively in populations with chronic low back pain. According to contemporary psychometric theory, measurement properties should be established in the specific population in which an instrument is intended to be used because validity and reliability are characteristics of the scores obtained in a particular context rather than inherent properties of the questionnaire itself [14,15,16]. Individuals with neck pain may differ from those with low back pain in symptom characteristics, functional limitations, psychosocial responses, and pain-related beliefs. Consequently, evidence obtained in low back pain populations cannot be assumed to generalize to individuals with neck pain. Therefore, we aimed to examine the measurement properties of the Arabic PSEQ in individuals with neck pain. Specifically, this study examined the internal consistency, structural validity, construct validity, measurement error, and test–retest reliability of the Arabic PSEQ. The Arabic PSEQ was hypothesized to have a one-factor structure, sufficient internal consistency, sufficient construct validity, acceptable measurement error, and sufficient test–retest reliability.

## 2. Materials and Methods

### 2.1. Study Design

This study used a prospective cohort study with two testing time points. The study design and analyses were guided by patient-reported outcome measures and internationally accepted methodological standards [14,17].

### 2.2. Setting and Participants

Participants were recruited from four hospitals in Riyadh, Saudi Arabia (King Khalid University Hospital, King Fahad Medical City, Kingdom Hospital, and King Abdullah bin Abdulaziz University Hospital). The Institutional Review Boards of the participating sites approved this study (approval no. R0-2023-E-002) (approval no. E-23-7671). A convenience sampling approach was used, whereby consecutive eligible patients attending the physical therapy clinics were invited to participate. Written informed consent was provided by all participants before participation.

To participate, individuals had to be at least 18 years old, be referred to physical therapy for neck pain as the primary complaint and be able to read and understand Arabic sufficiently to complete self-administered questionnaires independently. Participants were excluded if they had systemic diseases, cardiopulmonary or neurological conditions resulting in functional limitations, a history of spinal fracture, or previous cervical spine surgery.

### 2.3. Procedures

At their initial physical therapy visit, participants completed a battery of self-reported outcome measures including the PSEQ, Neck Disability Index (NDI), Numeric Pain Rating Scale (NPRS), and RAND 36-Item Health Survey (RAND-36). Participants also completed the same questionnaires for the second time within one week, prior to meaningful clinical change. Participants received a maximum of one physical therapy treatment session between the initial and retest assessments. At the second assessment, the perceived change in neck condition was also quantified using the Global Rating of Change (GRC) scale.

### 2.4. Outcome Measures

#### 2.4.1. Pain Self-Efficacy Questionnaire (PSEQ)

The PSEQ “[Pain Self-Efficacy Questionnaire] contact information and permission to use: Mapi Research Trust, Lyon, France, https://eprovide.mapi-trust.org” (accessed on 1 July 2026) is a 10-item questionnaire assessing the degree of confidence individuals have in performing activities in spite of pain [7]. Items are rated on a 0 to 6 scale (0 = not at all confident to 6 = completely confident), resulting in a 0 to 60 sum score with higher scores indicating greater pain self-efficacy. The current body of evidence supports sufficient PSEQ reliability, construct validity, and responsiveness in musculoskeletal pain populations [12]. Sufficient reliability and validity have also been established for the Arabic PSEQ, but only for individuals with chronic low back pain [13,18].

#### 2.4.2. Neck Disability Index (NDI)

The NDI is a neck-specific instrument assessing neck-related disability across 10 aspects [19] “[Neck Disability Index] contact information and permission to use: Mapi Research Trust, Lyon, France, https://eprovide.mapi-trust.org”. Items are rated from 0 to 5, yielding a total sum score presented as 0 to 100 percent. Higher scores indicate greater neck-related disability. The Arabic NDI has established validity and reliability in individuals with neck pain [20].

#### 2.4.3. RAND 36-Item Health Survey (RAND-36)

RAND-36 evaluates health-related quality of life in eight domains, including emotional well-being (mental health) and physical functioning [21]. Scores are presented on a 0–100 scale, with high scores representing better perceived health status. The Arabic RAND-36 has demonstrated good psychometric properties [22].

#### 2.4.4. Numeric Pain Rating Scale (NPRS)

The intensity of neck pain was quantified using the NPRS, an 11-point scale (0–10) with higher scores reflecting greater neck pain intensity. The NPRS has shown sufficient reliability and validity in individuals with neck pain [23]. A previous study has established support for the reliability and validity of the Arabic NPRS [24].

#### 2.4.5. Global Rating of Change (GRC)

Perceived neck condition change between assessment sessions was evaluated using the GRC. The GRC was scored from −5 (very much worse) to +5 (very much better). This measure is commonly used to classify individuals as unchanged or changed in studies evaluating reliability and responsiveness [25]. Individuals who reported no change (GRC score of 0), a tiny bit better, almost the same (GRC score of 1) or a tiny bit worse, almost the same (GRC score of −1) were considered in our study to have no or negligible change in their neck condition.

### 2.5. Statistical Analysis

#### 2.5.1. Structural Validity

Exploratory factor analysis (EFA) was used to test the structural validity of the PSEQ. Although previous studies have generally supported a unidimensional structure of the PSEQ, evidence regarding its dimensionality specifically in individuals with neck pain remains limited [26,27], and the dimensionality of the Arabic PSEQ has not previously been examined in this population. Therefore, EFA was selected to empirically examine the underlying factor structure without imposing a predefined measurement model. Given the ordinal nature of the PSEQ response options, EFA was conducted using weighted least squares estimation based on a polychoric correlation matrix, which provides more accurate estimates than Pearson correlations when analyzing Likert-type data [28,29]. Prior to factor extraction, Bartlett’s test of sphericity and the Kaiser–Meyer–Olkin (KMO) measure of sampling adequacy were evaluated to determine sufficiency of the data for factor analysis. Factor retention was determined using parallel analysis, which compares observed eigenvalues with those derived from randomly generated datasets and is considered one of the most robust methods for factor retention [30,31]. Items were considered to have meaningful loadings if they demonstrated a loading of ≥0.32, while cross-loadings were examined to ensure a clear and interpretable factor structure [32].

#### 2.5.2. Internal Consistency

Cronbach’s alpha was used to assess internal consistency, along with the assessment of changes in Cronbach’s alpha with the removal of individual items [15]. Item-rest correlations were also assessed.

#### 2.5.3. Test–Retest Reliability and Measurement Error

The intraclass correlation coefficient (ICC_2,1_) derived from a two-way random-effects model was used to assess the PSEQ test–retest reliability [15,33]. Standard error of measurement ($SEM=SD\times \sqrt{1-ICC};\;SD=pooled\;standard\;deviation$) and the minimal detectable change at the 95% confidence level (MDC_95_ = $1.96\times SEM\sqrt{2})$ were used to determine the PSEQ measurement error [15,33]. Agreement between repeated measurements was also assessed using a Bland–Altman plot with 95% limits of agreement [34]. Only individuals who reported no or negligible change in their neck condition (GRC scores ranging from −1 to +1) between the two PSEQ administrations were included in the test–retest reliability and measurement error analyses.

#### 2.5.4. Construct Validity

Testing a priori hypotheses regarding correlations between PSEQ scores and comparator instruments was implemented to examine the construct validity of the PSEQ. It was hypothesized that PSEQ would demonstrate moderate negative correlation with NDI and NPRS scores (r = −0.30 to −0.70), moderate positive correlations with RAND-36 physical functioning and RAND-36 emotional well-being (r = 0.30 to 0.70). PSEQ was also hypothesized to demonstrate a higher correlation (by at least 0.1) with NDI than NPRS. These hypotheses were based on theoretical expectations regarding the relationship between pain self-efficacy, disability, physical and emotional aspects of quality of life, and pain intensity. Pearson’s correlation coefficients were used to examine the predetermined correlational hypotheses with a 95% confidence interval estimated using bootstrapping. Confirmation of the predefined hypotheses was based on the direction and magnitude of the observed correlation coefficients rather than statistical significance; therefore, adjustment of *p*-values for multiple comparisons was not used for hypothesis confirmation.

### 2.6. Sample Size Estimation

Sample size considerations were guided by the COnsensus-based Standards for the selection of health Measurement INstruments (COSMIN) recommendations [35]. For factor analysis, a sample size of 60 participants (6 participants per item) was considered the minimum acceptable sample size. For construct validity, reliability, internal consistency, and measurement error, the minimum acceptable sample size was considered to be 50 participants.

## 3. Results

### 3.1. Participant Characteristics

The study included 118 individuals with neck pain (Table 1). Most of the participants reported chronic symptoms and radiating pain. Detailed demographic and clinical characteristics are presented in Table 1. Summary statistics for the PSEQ and other comparable measures are presented in Table 2. No missing responses were observed in any of the PSEQ items. Only two participants (1.7%) obtained the maximum PSEQ score, and no participant obtained the minimum score.

### 3.2. Structural Validity

**Figure 1:**
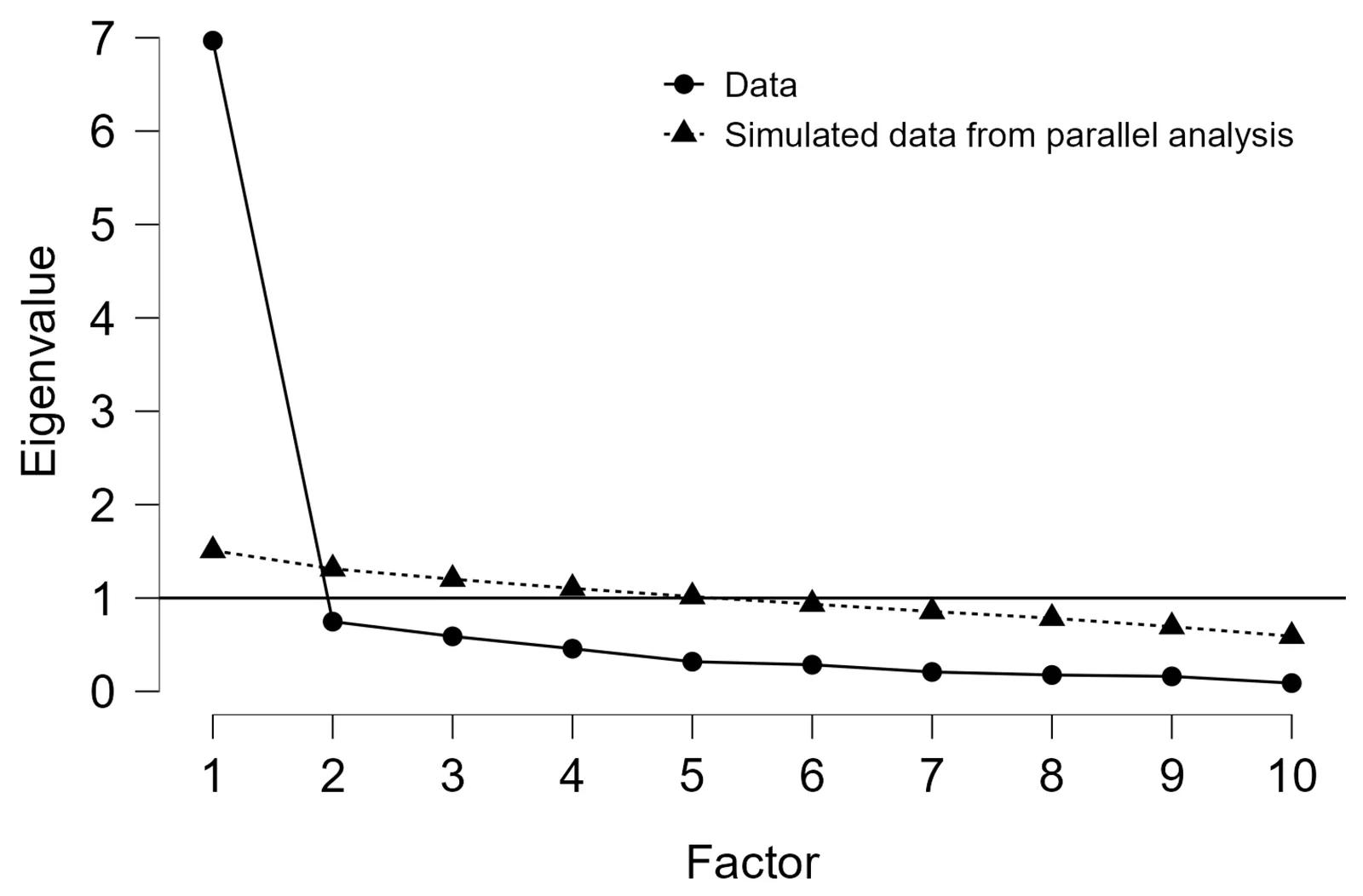
Scree plot with parallel analysis supporting PSEQ one-factor structure.

The results of the Kaiser–Meyer–Olkin (KMO) measure (0.919) and Bartlett’s test of sphericity (χ^2^(45) = 1084.73, *p* < 0.001) supported the suitability of the data for factor analysis. EFA supported the PSEQ unidimensional structure. Parallel analysis indicated retention of a single factor, as only the first observed eigenvalue (6.97) exceeded the corresponding simulated eigenvalue (1.51) (Figure 1). Most of the total variance (66.4%) was accounted for by the single factor, with all PSEQ items demonstrating strong factor loadings (0.72 to 0.89) (Table 3).

### 3.3. Internal Consistency

The PSEQ had Cronbach’s alpha of 0.94 (95% CI: 0.92–0.96), suggesting excellent internal consistency. Deletion of any individual item did not meaningfully change reliability, with Cronbach’s alpha ranging from 0.93 to 0.94 following deletion of individual items (Table 3). Item-rest correlations were uniformly high across the scale, ranging between 0.66 and 0.84, confirming that each individual item is highly representative of the overall pain self-efficacy construct (Table 3).

### 3.4. Test–Retest Reliability and Measurement Error

**Figure 2:**
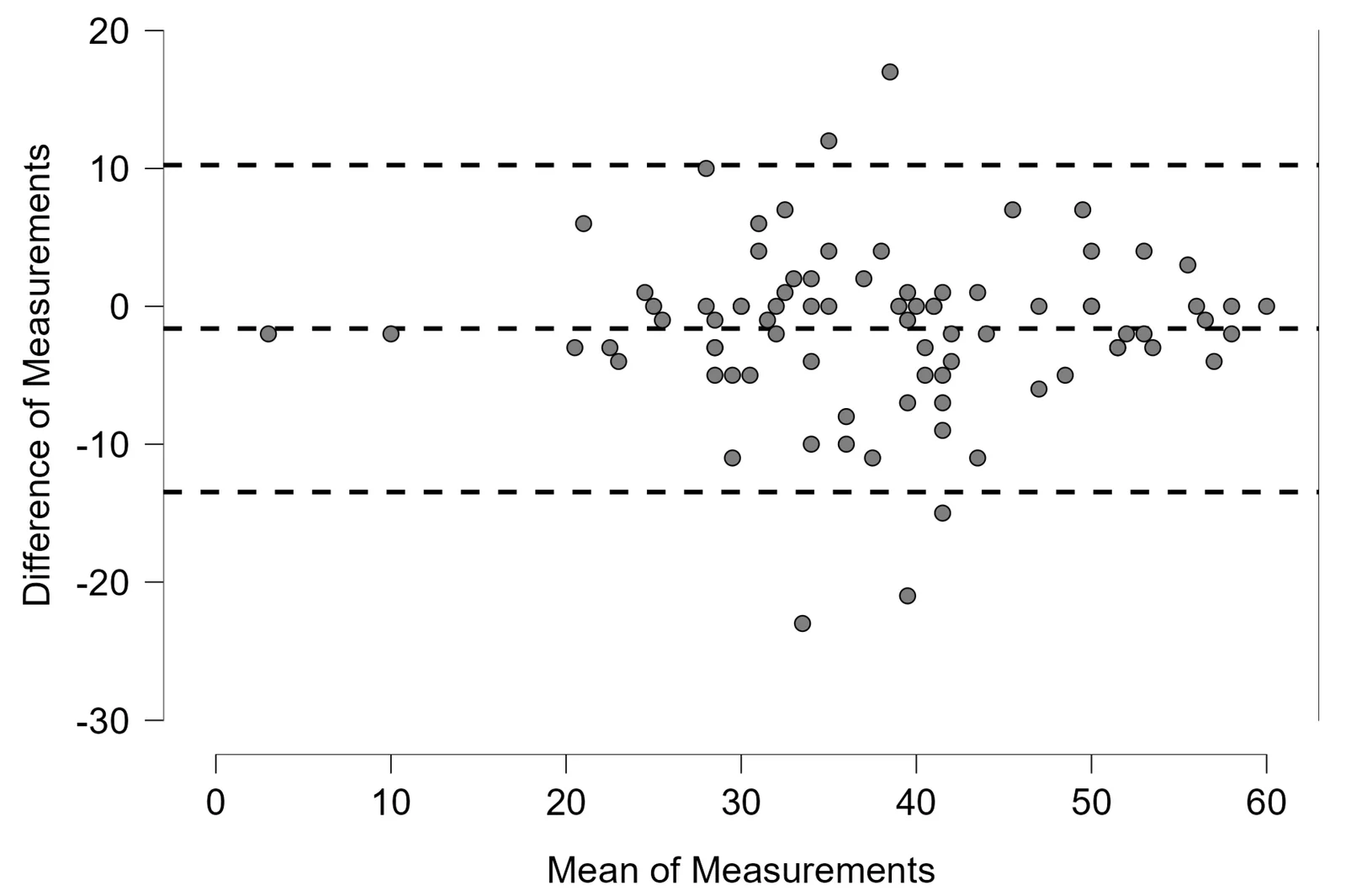
Bland–Altman plot showing agreement between PSEQ test and retest scores. The middle-dashed line represents the mean difference between test and retest scores while the upper and lower dashed line represent the 95% limits of agreement.

Of the 118 participants, 113 completed the retest assessment. Among those completing the retest, GRC scores were −3 in 1 participant, −2 in 4, −1 in 6, 0 in 28, +1 in 49, +2 in 8, +3 in 9, and +4 in 8. Based on the predefined criterion, 83 participants (73.5%) reported no or negligible change in their neck condition (GRC scores from −1 to +1) and were included in the test–retest reliability and measurement error analyses, whereas 30 participants (26.5%) were classified as changed and excluded from these analyses. The median interval between the initial and retest assessments was 4 days (IQR: 2–7 days). The PSEQ demonstrated good reliability, with an ICC_2,1_ of 0.85 (95% CI: 0.78–0.90). Measurement error analysis yielded a SEM of 4.38 and MDC_95_ of 12.14. Figure 2 displays the Bland–Altman analysis with a small mean difference (−1.61), indicating minimal systematic bias. The 95% limits of agreement extended from −13.47 to 10.24, suggesting moderate variability at the individual level. No evidence of proportional bias was observed.

### 3.5. Construct Validity

Construct validity was evaluated using predefined hypotheses regarding correlations with comparator instruments. As hypothesized, the PSEQ demonstrated a negative moderate correlation with the NDI, and positive moderate correlations with RAND-36 physical functioning and emotional well-being (Table 4). The PSEQ demonstrated a weaker-than-hypothesized negative correlation with NPRS (Table 4).

## 4. Discussion

Our study presented the first assessment of the measurement properties of the Arabic PSEQ in individuals with neck pain. Overall, the findings provided evidence supporting the internal consistency, structural validity, construct validity, and test–retest reliability of the instrument. Consistent with our predefined hypotheses, PSEQ unidimensionality was supported, internal consistency was excellent, test–retest reliability was good, and the majority of our construct validity hypotheses were confirmed.

Findings of the present study support the unidimensional structure of the PSEQ in individuals with neck pain. These findings are highly consistent with the theoretical framework proposed by Nicholas [7], who developed the PSEQ as a unidimensional measure of confidence in functioning despite pain. Consistent with our results, a systematic review supported the unidimensionality of the PSEQ in individuals with various musculoskeletal disorders [12]. Similarly, studies published after the systematic review also supported the unidimensionality of the PSEQ using different versions and in different populations [36,37]. Consistent with that, the unidimensionality of two Arabic PSEQ versions has also been reported previously, but these studies involved individuals with chronic low back pain rather than individuals with neck pain as reported in the current study [13,18]. Although previous studies have supported PSEQ unidimensionality, only two prior studies have examined its dimensionality specifically in individuals with neck pain, using the Italian and Hindi versions [26,27]. The present study extends this evidence by providing the first evaluation of the dimensionality of the Arabic PSEQ in individuals with neck pain. Confirmation of a one-factor structure supports the use of a single total score reflecting a single latent construct, namely pain self-efficacy, when interpreting the Arabic PSEQ in individuals with neck pain.

The Arabic PSEQ showed excellent internal consistency (Cronbach’s alpha = 0.94). Item-rest correlations ranged from 0.66 to 0.84, and deletion of individual items did not result in meaningful improvements in reliability. These findings indicate that all items contribute meaningfully to the assessment of pain self-efficacy and function cohesively as a unified scale. The internal consistency estimate observed in the current study is consistent with previous literature. The original English version demonstrated a Cronbach’s alpha coefficient of 0.92–0.93 [7]. Dube and associates reported the PSEQ internal consistency estimate to range from 0.79 to 0.95 across 20 studies with various musculoskeletal pain disorders [12]. Our findings are particularly comparable to those reported for the Arabic PSEQ, with reported Cronbach’s alpha coefficients of 0.90 and 0.93 in individuals with chronic low back pain [13,18]. Studies that included only individuals with neck pain [26,27] have also reported sufficient internal consistency of the PSEQ close to what is reported in our study (0.96 and 0.88), further strengthening our findings.

The Arabic PSEQ exhibited good test–retest reliability (ICC of 0.85). This point estimate is further supported by having a lower limit of the 95% confidence interval greater than the commonly recommended threshold (0.7) for sufficient reliability [14]. Our finding indicates that the questionnaire provides stable measurements over time when patients perceive no or negligible change in their condition. The reliability coefficient observed in our study is consistent with previous studies. The weighted average test–retest reliability coefficient across ten studies with various musculoskeletal pain disorders was reported to be 0.86 (range:0.75–0.93) [12]. This weighted average ICC is very similar to the reported ICC in the current study, providing support for our test–retest reliability estimate. The previous Arabic validation studies conducted in individuals with chronic low back pain also reported sufficient test–retest reliability (ICC of 0.79 and 0.97), with our reported estimate falling within these reported estimates [13,18]. One prior study has examined the PSEQ test-retest reliability specifically in individuals with neck pain, with a reported ICC of 0.90 [27]. Collectively, these findings provide support for the reproducibility of PSEQ scores and reinforce its suitability for repeated assessments in individuals with neck pain.

SEM and MDC_95_ were used to estimate the measurement error of the PSEQ. Clinically, the observed MDC_95_ of 12.14 points indicates that a change greater than approximately 12 points is required to be confident, at the 95% level, that the observed change exceeds measurement error rather than reflecting random variation in repeated measurements. An MDC value smaller than the scale’s minimal important change value is suggested to reflect acceptable measurement error [14,38]. Because a minimal important change value has not yet been established for the Arabic PSEQ in individuals with neck pain, it remains unknown whether an MDC of 12.14 points reflects acceptable measurement error. The MDC reported here reflects 20% of the PSEQ score range, and this magnitude of measurement error is considered by some to be at the upper end of what is considered an acceptable level of measurement error [39]. The MDC reported in the current study lies within the range of MDC values (14.6 and 5.93 points) reported previously for the Arabic PSEQ in patients with chronic low back pain [13,18]. The weighted average MDC value of three studies including patients with chronic pain and chronic low back pain has been reported to be 11.52 (range: 3.41–15.69) [12]. This weighted average MDC is very close to the reported MDC in the current study, providing confidence in the level of measurement error estimated in our study.

Construct validity of the Arabic PSEQ was supported given that four out of five (80%) predefined hypotheses were supported. As hypothesized, the PSEQ demonstrated a moderate correlation in the expected direction with measures of disability and physical functioning (NDI, RAND-36 physical functioning). This moderate relationship is consistent with theoretical expectations and previous literature. In individuals with idiopathic and chronic neck pain, PSEQ demonstrated a moderate negative correlation with NDI (r = −0.57; r = −0.48) [26,27], consistent with our findings. Additionally, the previous literature corroborated our reported positive moderate correlation between the PSEQ and RAND-36 physical functioning, with reports of positive moderate correlations between the PSEQ and the SF-36 and SF-12 physical components (range: 0.38–0.52) in individuals with various musculoskeletal pain disorders [12].

In the current study, the PSEQ demonstrated a significant negative correlation with pain intensity (NPRS); however, the observed point estimate (r = −0.25) fell slightly below the predefined magnitude (r = −0.30 to −0.70) and therefore did not meet the a priori criterion for hypothesis confirmation. Nevertheless, the 95% confidence interval (−0.41 to −0.09) included values within the hypothesized range, indicating that the observed estimate remains reasonably compatible with the theoretically expected relationship. Previous studies have reported weak-to-moderate negative correlations between PSEQ scores and pain intensity measures, typically ranging from −0.05 to −0.50 [7,12,13,26,27]. The correlation observed in the current study is therefore highly consistent with previous evidence. Pain self-efficacy beliefs influence individuals’ willingness to engage in activities despite pain and therefore were expected to be more strongly related to disability than to pain intensity itself [7]. Previous studies involving individuals with neck pain have reported stronger correlations of pain self-efficacy with disability than with pain intensity [26,27] consistent with our findings.

Limitations of the present study should be acknowledged. Although participants were recruited from four hospitals, all recruitment sites were located in Riyadh, Saudi Arabia, which may limit the generalizability of the findings to Arabic-speaking populations in other geographic or sociocultural settings. In addition, most participants had chronic neck pain, and the findings may therefore not fully generalize to individuals with acute or subacute neck pain. The numbers of potentially eligible individuals who declined participation or were excluded before enrollment were not prospectively recorded, limiting a complete assessment of potential selection bias. Responsiveness and interpretability, including determination of the scale’s minimal important change threshold, were not evaluated and should be examined in future studies. Finally, measurement invariance analyses were not performed and should be considered in future research to further evaluate the structural stability of the instrument across demographic and clinical subgroups. Although EFA was considered appropriate for the first evaluation of the dimensionality of the Arabic PSEQ in individuals with neck pain, future studies should use confirmatory factor analysis in independent samples to confirm the one-factor structure identified in the present study. Clinical stability was determined using the self-reported GRC, with scores from −1 to +1 classified as representing no or negligible change. Although these response options indicate that participants perceived their condition as almost the same, very small changes between assessments cannot be completely excluded and may have influenced the test–retest reliability estimate. This study has several strengths. It followed COSMIN recommendations for evaluating measurement properties of patient-reported outcome measures, included an adequate sample size for all analyses, and used robust psychometric methods including an appropriate EFA estimator for ordinal data with polychoric correlations and parallel analysis. Furthermore, multiple measurement properties were evaluated within the same cohort of individuals with neck pain.

## 5. Conclusions

The Arabic PSEQ demonstrated excellent internal consistency, one-factor structure, good test–retest reliability, sufficient construct validity, and acceptable measurement error in individuals with neck pain. Overall, the findings provide support for the use of PSEQ as a valid and reliable instrument for the assessment of pain self-efficacy among Arabic-speaking individuals with neck pain. Future studies should evaluate responsiveness, interpretability, and measurement invariance to further strengthen the evidence supporting its use in clinical practice and research.

## Figures and Tables

**Table 1:** Characteristics of participants (N = 118).

Variable	Mean ± SD or *N* (%)
Age (year)	44.07 ±12.39
Sex	
Male	46 (38.98)
Female	72 (61.02)
Height (m)	1.64 ± 0.10
Mass (Kg)	74.79 ±12.59
Body mass index (Kg/m^2^)	27.90 ± 4.27
Neck pain duration	
<3 weeks	7 (5.93)
3–12 weeks	16 (13.56)
>12 weeks	95 (80.51)
Radiating pain	
Yes	88 (74.58)
No	30 (25.42)
Educational level	
Elementary school	5 (4.2)
Middle school	2 (1.7)
High school	12 (10.2)
Undergraduate degree	81 (68.6)
Postgraduate degree	17 (14.4)
Missing	1 (0.8)

**Table 2:** Outcome measures (N = 118).

Variable	Mean ± SD
PSEQ (0–60)	37.48 ± 11.71
NDI (0–100)	38.76 ± 13.89
NPRS (0–10)	6.05 ± 1.98
RAND-36 physical functioning (0–100)	63.47 ± 20.22
RAND-36 emotional well-being (0–100)	55.76 ± 17.54

NDI = Neck disability index; PSEQ = Pain Self-Efficacy Questionnaire; NPRS = Numeric pain rating scale; RAND-36 = RAND 36-Item Health Survey.

**Table 3:** PSEQ Item-level analyses.

Item	Factor Loading	Cronbach’s α (If Item Dropped)	Item-Rest Correlation
Item 1	0.839	0.933	0.794
Item 2	0.731	0.939	0.683
Item 3	0.791	0.936	0.730
Item 4	0.855	0.933	0.810
Item 5	0.738	0.939	0.671
Item 6	0.808	0.935	0.764
Item 7	0.720	0.940	0.661
Item 8	0.881	0.932	0.822
Item 9	0.886	0.931	0.840
Item 10	0.874	0.932	0.828

PSEQ = Pain Self-Efficacy Questionnaire.

**Table 4:** Correlations between PSEQ scores and comparator instruments.

	r	*p*	95% CI Lower Limit	95% CI Upper Limit
NDI	−0.48	<0.001	−0.61	−0.33
RAND-36 physical functioning	0.38	<0.001	0.20	0.54
RAND-36 emotional well-being	0.42	<0.001	0.23	0.58
NPRS	−0.25	0.007	−0.41	−0.09

NDI = Neck disability index; PSEQ = Pain Self-Efficacy Questionnaire; NPRS = Numeric pain rating scale; RAND-36 = RAND 36-Item Health Survey.

## Data Availability

The data presented in this study are not publicly available due to privacy and ethical restrictions but are available from the corresponding author upon reasonable request.
